# Exceptional longevity and potential determinants of successful ageing in a cohort of 39 Labrador retrievers: results of a prospective longitudinal study

**DOI:** 10.1186/s13028-016-0206-7

**Published:** 2016-05-11

**Authors:** Vicki Jean Adams, Penny Watson, Stuart Carmichael, Stephen Gerry, Johanna Penell, David Mark Morgan

**Affiliations:** 1 Vet Epi, White Cottage, Dickleburgh, Norfolk IP21 4NT UK; 2 Department of Veterinary Medicine, University of Cambridge, Madingley Road, Cambridge, CB3 OES UK; 3 University of Surrey, Vet School Main Building, Daphne Jackson Road, Guildford, GU2 7AL UK; 4 Nuffield Department of Orthopaedics, Centre for Statistics in Medicine, Rheumatology and Musculoskeletal Sciences, University of Oxford, Oxford, OX3 7LD UK; 5 School of Veterinary Medicine, Faculty of Health and Medical Sciences, University of Surrey, Vet School Main Building, Daphne Jackson Road, Guildford, GU2 7AL UK; 6 Spectrum Brands Schweiz GmbH, Stationsstrasse 3, Brüttisellen, 8306 Zurich, Switzerland

**Keywords:** Ageing, Exceptional longevity, Healthspan, Body weight, Sarcopenia, Lean body mass, Body fat mass, Nutrition, Husbandry, Healthcare

## Abstract

**Background:**

The aim of this study was to describe the longevity and causes of mortality in 39 (12 males, 27 females) pedigree adult neutered Labrador retrievers with a median age of 6.5 years at the start of the study and kept under similar housing and management conditions. Body condition score was maintained between two and four on a 5-point scale by varying food allowances quarterly. The impact of change in body weight (BW) and body composition on longevity was analysed using linear mixed models with random slopes and intercepts.

**Results:**

On 31 July 2014, 10 years after study start, dogs were classified into three lifespan groups: 13 (33 %) Expected (≥9 to ≤12.9 years), 15 (39 %) Long (≥13 to ≤15.5 years) and 11 (28 %) Exceptional (≥15.6 years) with five still alive. Gender and age at neutering were not associated with longevity (P ≥ 0.06). BW increased similarly for all lifespan groups up to age 9, thereafter, from 9 to 13 years, Exceptional dogs gained and Long-lifespan dogs lost weight (P = 0.007). Dual-energy x-ray absorptiometer scans revealed that absolute fat mass increase was slower to age 13 for Long compared with Expected lifespan dogs (P = 0.003) whilst all groups lost a similar amount of absolute lean mass (P > 0.05). Percent fat increase and percent lean loss were slower, whilst the change in fat:lean was smaller, in both the Exceptional and Long lifespan compared with Expected dogs to age 13 (P ≤ 0.02). Total bone mineral density was significantly lower for Expected compared to Exceptional and Long lifespan dogs (P < 0.04).

**Conclusions:**

This study shows that life-long maintenance of lean body mass and attenuated accumulation of body fat were key factors in achieving a longer lifespan. The results suggest that a combination of a high quality plane of nutrition with appropriate husbandry and healthcare are important in obtaining a greater than expected proportion of Labrador retrievers living well beyond that of the expected breed lifespan: 89.7 % (95 % CI 74.8–96.7 %) dogs were alive at 12 years of age and 28.2 % (95 % CI 15.6–45.1 %) reaching an exceptional lifespan of ≥15.6 years.

## Background

For the domesticated dog (*Canis lupus familiaris*), changes observed through ageing can be seen as good (e.g. improved obedience), bad (e.g. dental disease) or inconsequential (e.g. greying of the muzzle) with respect to their viability and survival. Physiological changes that may be important biomarkers of ageing in dogs include increasing body fat, reducing lean body mass (of which lean muscle mass is an important component), periodontal disease, osteoarthritis, reduced renal or cardiac function, changes to the endocrine system (including insulin and glycaemic control), cognitive and behavioural changes and the development of neoplastic disease [1–4].

The goal for today’s biogerontologists is to extend human healthspan, defined as the years in which an individual is generally healthy and free from serious disease, alongside increasing longevity; this goal can also be applied to our companion animals [5]. In the domestic dog, reported average longevity estimates for all breeds combined have varied between 10.0 and 12.0 years, depending on the population studied [6–8]. As part of the current project, an external panel of veterinary and academic experts was convened to independently and objectively define an average lifespan for Labrador retrievers based on median/mean age at death reported in published research and from a proprietary service dog database as well as their professional knowledge and/or personal experience. Upon review of the body of evidence available from 1999 to 2013, a consensus was reached that the typical lifespan of Labrador retrievers was 12 years of age (Table 1). The domesticated dog represents an exceptional range of phenotypic morphology with breeds varying in weight by two-orders of magnitude [9]. Canine life expectancy and body mass are inversely correlated with size explaining 40–44 % of the variance in age at death [8, 9]; small breeds typically live much longer than large breeds [8, 10–12]. It is not clear what effect neutering has on longevity as one study reported that neutering was associated with increased longevity for females but not males in the UK [6], whilst neutered males outlived entire males among US military dogs [13]. Another study has shown that neutering was strongly associated with an increase in lifespan as well as a decreased risk of death from some causes, such as infectious disease, but an increased risk of death from others, such as cancer [14]. The discrepancies might be related to the age of neutering, however, there is a lack of information on this.

**Table 1:** Evidence used in consensus for ‘Typical’ Labrador retriever lifespan based on reported ages at death with reference numbers in square brackets

Reference material	Country	#Dogs	Median lifespan, years
Assistance dog database^a^	US	498	12.0
Lawler et al. [2]	US	48	‘Restricted’ group: 13.0
Kealy et al. [19]			‘Control’ group: 11.2
Adams et al. [8]	UK	574	12.25
O’ Neill et al. [12]	UK	418	12.5
Michell [6]	UK	328	12.6
Proschowsky et al. [7]	Denmark	199	10.5
Typical lifespan of Labrador retrievers^b^			12.0

^a^Canine companions for independence (Santa Rosa, CA, USA)

^b^Consensus age provided by Jan Bellows, DVM, DAVDC, DABVP, FAVD; Carmen M. H. Colitz, DVM, Ph.D., DACVO, Donald Ingram, Ph.D.; Stanley L. Marks, BVSc, Ph.D., DACVIM, DACVN; Sherry L. Sanderson, DVM, Ph.D., DACVIM, DACVN; Julia Tomlinson, BVSc, Ph.D., DACVS, CCRP, CVSM

With the large disparity in longevity of individual dog breeds, the challenge is to understand the biological mechanisms that underlie these apparent differences [15]. The term ‘exceptional longevity’ has be used to describe both groups of, and individual, dogs that live 30 % longer than is expected for their breed’s typical or average lifespan [16]. Dogs that live for a longer period than their anticipated lifespan appear to demonstrate an ability to delay the onset of life-threatening diseases [16–18]. A previous study of 24 pairs of Labrador retriever littermates from 7 litters showed that lifetime calorie restriction resulted in a 1.8 year longer median lifespan in the ‘Restricted’ group fed 25 % less than the ‘Control’ group (P = 0.004) [19].

The longitudinal study reported here is a continuation of efforts to understand the biology of ageing and its valuable application to companion dogs. The aim of this study was to describe the longevity and causes of mortality in a cohort of purebred Labrador retrievers kept under similar housing and management conditions. Additionally, we wished to evaluate the impact of gender, age at neutering, and changes in body weight and body composition on longevity.

## Methods

### Background and animal selection

**Fig. 1:**
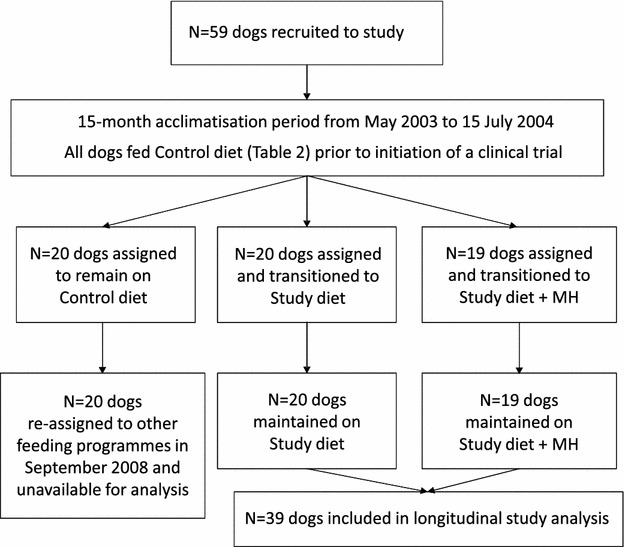
Flowchart of dogs recruited to the study and included in the analysis. *MH* mannoheptulose

The original study was designed as a clinical trial to test a novel energy restriction mimetic in the form of the dietary supplement mannoheptulose (MH). MH is a seven-carbon sugar derived from avocado that acts to reduce glycolysis via hexokinase inhibition and has been proposed as a calorie restriction mimetic that delivers anti-ageing and health promoting benefits of calorie restriction without reducing food intake [20, 21]. Initially, three groups of dogs were formed from a cohort of 59 Labrador retrievers after a 15-month acclimatisation period from May 2003 to 15 July 2004 (Fig. 1). During the acclimatisation period, fasting blood glucose and insulin were measured and the dogs were allocated to treatment groups using stratified randomisation based on these levels. The study design was approved by the Institutional Animal Care and Use Committee (IACUC) of Procter and Gamble (P&G) Pet Care (Mason, OH, USA).^1^ The accommodation facility where the dogs were housed, the P&G Pet Care Pet Health and Nutrition Center in Lewisburg, OH, USA, was accredited by the Association for Assessment and Accreditation of Laboratory Animal Care. After the initiation of the clinical trial an independent International Animal Welfare Advisory Board also had input and recommendations principally on the dogs’ behaviour and enrichment interventions. This board made unannounced visits to the centre and their reports were made publically available.^2^

At the start of the original clinical trial (16 July 2004) one group was fed a control diet (n = 20, Table 2), one group was fed a study diet matrix (n = 20, Table 2) and the third group was fed the same study diet matrix with the inclusion of avocado juice extract with a concentration of <0.10 % as a source of biologically available mannoheptulose (MH) (n = 19) [22]. As a result of an internal business decision, the group of dogs that had been fed the control diet were released from the clinical trial and entered other feeding programmes in September 2008. The feeding of the two other groups continued as before with the same allocated study diet with or without MH (Fig. 1). Statistical analysis in June 2013 showed no significant effect of the diet on longevity detected between the 19 dogs that received the study diet and MH and the 20 dogs that were just fed the study diet; therefore, the data for these dogs were combined for the longevity analysis in this study (Fig. 1). For the remainder of this paper we report only on these 39 dogs.

**Table 2:** General ingredient and nutrient composition of the control and study diets

Ingredient composition			Analysis %		
Control diet^a^	Study diet^b^		Nutrient^f^	Control	Study
Maize	Chicken meal	Egg product	Protein (%)	25.1	24.7
Chicken by-product meal	Chicken by-product meal	Brewer’s yeast	Fat (%)	13.8	15.0
Maize gluten meal	Maize	FOS^c^	Fibre (%)	2.1	2.1
Soybean meal	Sorghum	sHMP^d^	Ash (%)	5.3	7.0
Animal fat	Barley	Linseed	Moisture (%)	7.8	7.5
Palatant	Chicken fat	l-carnitine	Vitamin E (IU/kg)	163	328
Minerals	Fishmeal	Minerals	β-carotene (ppm)	2.9	39
Vitamins	Palatant	Vitamins	GE^g^ (kcal/kg)	4716	4695
	Beet pulp	Other^e^	6:3 fatty acids^h^	19:1	9:1

^a^The control diet was nutritionally complete and balanced and formulated to be representative of a mid-tier adult dog food product

^b^Study diet was formulated to contain the same nutritional technologies found in the Eukanuba nutritional matrix for senior dogs (Eukanuba^®^ Senior Maintenance Dog Food. For the study period reported this product was owned and manufactured by Procter & Gamble, Cincinnati, Ohio, USA)

^c^Fructo-oligosaccharide

^d^Sodium hexametaphosphate

^e^Avocado juice concentrate (<0.10 %) was included in the diet matrix for 19 dogs

^f^Nutrient composition is actual laboratory analytical results expressed on as-fed basis

^g^Gross energy

^h^Ratio of dietary omega-6 fatty acids and omega-3 fatty acids

A total of 39 adult, neutered Labrador retrievers consisting of 12 males and 27 females recruited in early to mid-adulthood, between the ages of 5.3 and 8.5 years (mean age 6.7 years, median 6.5), were included in the current longitudinal study. All dogs were acquired from private breeders or United States Department of Agriculture-inspected provider. One breeder provided 33 (85 %) Labrador retrievers from breeding a total of 12 sires and 19 dams. For these 33 dogs, the husbandry (feeding and kennelling) and medical care (worming and vaccinations) was kept consistent for all the dogs from birth and through early adulthood prior to them being recruited for the study. Furthermore, these dogs were housed in groups of three (one male and two female) in large open paddocks that were 15 metres wide by 45 m long covered with gravel on a concrete base. Covered shelter was available (the internal housing used straw bedding) from the heat/cold and the dogs were all exposed to the same natural day/night light cycles. Each dog could hear and see their neighbours in the other paddocks. The breeder would rotate the males and females according to the breeding programme. After recruitment, 38 entire dogs were surgically neutered by the supervising veterinarian in 2003 prior to the start of the study period in July 2004 with only one dog, a male, that had been neutered at 4.4 years of age just prior to being recruited into the study in 2003. The mean and median age at neutering was 5.5 years (range 4.3–7.5 years).

### Animal husbandry and veterinary care

Dogs were housed in a kennel system that include indoor kennels and outdoor runs. Both the indoor kennels and outdoor runs were cleaned and disinfected on a regular basis, and all had size-appropriate dog toys that were cleaned and interchanged regularly to provide environmental enrichment. The temperature of the indoor kennel was kept at approximately 22 °C (range 18–25 °C) with a relative humidity of 50 % (range 40–70 %). Air flow through the kenneling facility was maintained with approximately 15 fresh air changes per hour (range 10–30). Natural light was provided by large rectangular windows that were positioned above each of the individual indoor kennels and ran on both sides of an open corridor and for the entire length of the facility. The indoor kenneling area also had an automated lighting system that controlled the light/dark cycle with 12 h on from approximately 06.00–18.00 and 12 h off (Invensys system, Invensys Operations Management Company, Plano, TX, USA).

Each dog was groomed every 2 weeks (brushing, nail trimming, examination for parasites and skin lesions) and bathing was done quarterly. Daily socialisation of all dogs took place (20 min) with a qualified animal welfare specialist and additionally social groups of three to six dogs were exercised outdoors daily (30 min minimum) in a large gravel lined or grass exercise area. Furthermore, compatible groups of dogs had 24-h access to each other through their partially covered run that interconnected neighbouring kennels.^3^ Equipment designed to provide environmental enrichment (e.g. agility course apparatus, wading pools, a variety of dog toys and shaded areas) was provided in the large areas.

The preventative healthcare plan for each dog included faecal examinations annually for endoparasites alongside blood testing for heartworm. Heartworm prevention was given monthly (Interceptor^®^, Novartis Animal Health, US) and flea and tick treatment was given when required (Frontline Plus^®^, Merial) [23]. The vaccination routine consisted of adenovirus type 2, distemper, parvovirus, parainfluenza, (± leptospira given according to a health risk assessment) and an intranasal vaccine for Bordetella administered annually according to manufacturer’s recommendations (Fort Dodge Duramune Max 5/4L^®^; Shering-Plough Intra-Trac II ^®^Bordetella Vaccine). Vaccination against Rabies was given every three years (Fort Dodge RabVac^®^ 3TF). Oral health was evaluated with dental examinations performed every 6 months; standard dental prophylaxis/treatment (e.g. extractions, descaling and polishing under anaesthesia) was performed for each dog when necessary as recommended by the attending veterinarian. Standard physical examinations were undertaken annually and blood samples collected every 6 months for routine clinical assessment. Clinical parameters measured included complete blood cell counts, serum biochemistry and thyroid function. Aside from the regular veterinary examinations, each dog was monitored daily by their animal welfare specialist and the animal husbandry staff, and any health related concerns (e.g. medical, orthopaedic, oncologic) were brought to the attention of the attending healthcare staff. At this point the healthcare staff would initiate a regular quality of life assessment to monitor the dog’s health and to assess if it remained stable or declined.^4^ The case was then discussed by a group that was blinded to the identity of the dog. Decisions about medications and end-of-life issues were made by the group based on whether the quality of life was declining and the dog’s overall well-being was compromised (see footnote 4). The group consisted of the study director, several veterinarians within other business units of the company as well as other veterinarians from the pet care unit. The on-site attending veterinarian also was authorised to make an end-of-life decision if there was a rapid deterioration in any dog’s health.

### Diet and feeding

From May 2003 until the start of the study on 16 July 2004 all dogs spent 1 year on a nutritionally complete and balanced control diet which was formulated to be representative of a mid-tier adult dog food (Control diet, Table 2). This was to help acclimatise the dogs to their environment and to help adjust their body weight and body condition score so that they entered the study in 2004 with a body condition score (BCS) between two and four, based on a 5-point BCS [24]. This adaptation period also helped establish the individual food allowance required to maintain body weight with a BCS between two and four. Following this year of acclimation, the 39 dogs were transitioned onto a study diet formulated with the same nutritional technologies shown to help support the health and well-being of both adult and senior dogs (Study diet, Table 2). This dietary matrix was created to comprise the same nutritional components found in Eukanuba^®^ Senior Maintenance^5^ for large breed dogs but with a slightly lower protein (24.7 % ‘as fed’) and slightly higher fat (15 % ‘as fed’) level.

BCS was assessed by trained staff on a quarterly basis both during the adaptation year and during the study using a five-point scale. A score of 3 was considered ideal. A dog’s daily food allowance was changed if the quarterly BCS was not between the 2–4 range to avoid the extremes of thinness (BCS = 1) or obesity (BCS = 5) conditions. The maximum allowable change to an individual dog’s daily food allowance was ±50 g and this food amount was maintained until the next quarterly BCS assessment. The daily food allowance could also be changed by the supervising veterinarian for medical purposes.

The daily ration of food was weighed for each dog, divided into two equal portions, and offered in stainless steel food bowls inserted into rings located at the front of each indoor pen at 07:30 and 14:30 each day. Dogs were separated for feeding. Each dog was allowed 30 min to consume their food and food intake was recorded daily. Fresh water was constantly available using automatic adjustable water bowls mounted on the side of each housing unit.

### Body weight and composition

Body weight was measured every 2 weeks, BCS was evaluated quarterly and whole-body composition measures were obtained prior to the start of the study and then annually using a Dual-Energy X-ray Absorptiometer (DEXA scan) (Model Delphi-A, Serial No. 70852; Bedford, MA). For the DEXA scan, dogs were fasted for a minimum of 12 h prior to being sedated using a pre-anaesthetic combination of Acepromazine (PromAce^®^ Injectable, Fort Dodge, Fort Dodge, Iowa; 0.55 mg/kg intramuscular injection) and Atropine Sulfate (Med-Pharmex, Pomona, CA; 0.04 mg/kg subcutaneous injection). Dogs were then anaesthetised with Propofol administered via a secured intravenous catheter (Propoflo^®^, Abbot Labs, Chicago, IL; 7 mg/kg), intubated with an endotracheal tube and delivered 100 % oxygen. Routine anaesthetic monitoring was performed. Dogs were positioned in sternal recumbency with hind limbs extended caudally. A calibration was completed prior to each DEXA scan and measurements were taken using the whole body scanner (single-beam mode). After the scans, anaesthesia was discontinued and oxygen was continued for several minutes. Dogs were moved to a recovery cage and the endotracheal tube was removed once the swallowing reflex was regained. Whole-body measures obtained from the DEXA scans included total bone mineral density (BMD in grams), total bone mineral content (BMC in grams), total body mass (g), total fat mass (g), total lean mass (g), % body fat, % body lean and fat to lean ratio (determined as total fat mass/total lean mass).

### Statistical analysis

**Fig. 2:**
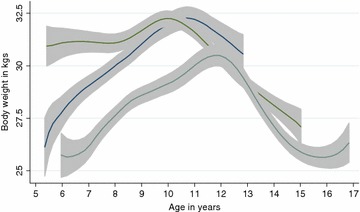
Polynomial smooth *plots* of average body weight (*lines*) with 95% confidence intervals (CI,* gray shaded areas*) by age for each longevity group of dogs. The starting point for each line is the average body weight for those dogs which were in the acclimatisation period at that age on 01 July 2004 just before the study started. The end point for each line is the average body weight for those dogs which died or were censored (N = 5 in the Exceptional group) at that age at the censor date of 31 July 2014. These *plots* show that the Expected dogs (*blue line*) started at a low weight, then put on about 1 kg/year until reaching a peak at 11 years of age and this was followed by a decline of ~1.7 kg in 2 years. The Long-lived dogs (*green line*) started at a higher weight, then stayed at a rather stable weight before showing an increased weight over to reach a peak at 10 years of age and then the weight declined at ~1 kg/year. The Exceptionally long-lived dogs (*gray*) started at the lowest weight (but at an older age) and they put on weight gradually to reach a peak at 12 years of age, then slowly declined to reach a low point at 16 years before putting on some weight again

Dogs were classified into three groups derived from tertiles of lifespan data as of 31 March 2014: Expected’ when they experienced a lifespan of ≥9 to ≤12.9 years, ‘Long’ when they experienced a lifespan between ≥13 and ≤15.5 years and ‘Exceptional’ when they achieved ≥15.6 years and beyond. The value of 15.6 for the Exceptional lifespan group is 30 % longer than the 12-year typical lifespan of the breed determined by the consensus group (Table 1) [16]. Average body weights and ages among the three longevity groups at the start of the longitudinal study were compared using analysis of variance with post hoc pairwise comparisons. Cross-tabulations and Chi square or Fishers Exact tests were used to compare proportions of dogs within various groups. Survival analysis, using Kaplan–Meier (KM) and Cox proportional hazards regression (Cox) models, was performed to examine the effect of potential predictors on time to death. Monthly body weights up to December 2013 and annual body composition data up to 13 years of age were analysed using linear mixed models with random effects for slopes and intercepts and a fixed effect for the lifespan grouping variable. The models allowed an intercept and slope to be estimated for each dog with the assumption that each endpoint response for a dog had a linear trajectory across age. Body weight against age presented by longevity category in a polynomial smooth plot did not show a linear trend but a curve that follows an inverted U shape (Fig. 2); therefore, a random coefficient model was used to compare the slopes as a measure of body weight change (kg/dog/year) for three groups: up to 9 years, 9–13 years and >13 years of age. An age cut-off point of 13 years was chosen for statistical analysis because all three lifespan groups were fully represented up to age 13. Statistical analysis was performed using commercial software.^6^ The level of significance for all statistical tests was set at *P* < 0.05.

## Results

### Longevity and cause of death

**Fig. 3:**
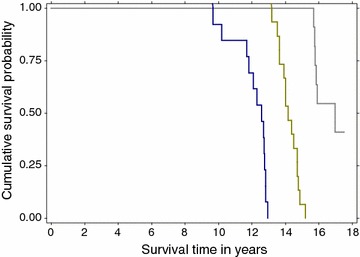
Kaplan–Meier survival plot for 39 Labrador retrievers in three lifespan groups. Expected (*blue*): 13 dogs; Long (*green*): 15 dogs; and Exceptional (*gray*): six deceased and five dogs remaining alive as of the July 31, 2014 censor date

As of July 2004, the dogs that were subsequently classified as experiencing an Exceptional lifespan were significantly older than the other dogs (P = 0.01) and there were no significant differences in body weight (P = 0.36, Table 3). The median time spent in this longitudinal study (from 16 July 2004 to death or the censor date for longevity estimation) by the 39 dogs was 7.43 years and this ranged from 2.54 to 10.04 years. As of the censor date of 31st of July 2014, the distribution of dogs based on lifespan groups was: Expected lifespan (n = 13), Long lifespan (n = 15) and Exceptional lifespan (n = 11 including 5 dogs that remained alive with a median age of 17.1 years, Table 4; Fig. 3). A total of 35 dogs (89.7, 95 % CI 75–97 %) were alive at 12 years of age and 11 dogs (28.2, 95 % CI 15.6–45.1 %) attained exceptional longevity, reaching or exceeding 15.6 years of age. The five exceptionally long-lived dogs that remained alive at the censor date were considered to be 3–4 years younger than their chronological age, based on overall condition, activity level and interactive behaviour as observed by several independent veterinarians and Labrador retriever enthusiasts who interacted with these dogs in June 2014. For the 34 dogs that passed away during the study the age at death ranged from 9.7 to 16.9 years with a median age of death of 13.6 years. Each of the lifespan groups had significantly different median survival time (MST) based on KM survival analysis (Table 4; Fig. 3). A larger proportion of female dogs reached an Exceptional age (female:male ratio = 10:1) compared with the Expected (7:6) and Long (10:5) lifespan groups although this was not a statistically significant difference (*P* = 0.1). Gender was not significantly associated with survival time (KM P = 0.06) or risk of death (Cox *P* = 0.07) and there was no effect of the age of neutering on the risk of death for females (Cox *P* = 0.2) or males (Cox *P* = 0.7).

**Table 3:** Mean ages and body weights for the three longevity groups of dogs in July 2004

Longevity category	N	Mean body weight (kg)	SD	Min–max	Mean age (years)	SD	Min–max
Expected (≥9 to ≤12.9 years)	13	29.4	4.6	20.2–37.40	6.5*	0.6	5.3–7.6
Long (≥13 to ≤15.5 years)	15	29.4	4.3	21.1–41.4	6.4^#^	0.9	5.4–7.9
Exceptional (≥15.6 years)	11	26.8	3.7	19.2–33.1	7.4*^#^	0.8	6.0–8.5

Means among longevity groups were compared using analysis of variance with post hoc pairwise comparisons; mean body weights were not significantly different (P = 0.36) whilst means within the age column that share an asterisk (*) or a hash (#) were significantly different (P = 0.01)

*N* number of dogs, *SD* standard deviation, *Min–max* range from minimum to maximum values

**Table 4:** Number of Labrador retrievers, age at death/censor date and median survival time with 95 % CIs

Longevity category		Descriptive statistics for age in years at death/censor date^a^			MST (95 % CI) in years from Kaplan–Meier analysis
		N (%)	Mean (SD)	Median (min–max)	
Expected ≥9 to ≤12.9	Deceased	13 (33 %)	12.08* (1.04)	12.58* (9.68–12.95)	12.44* (11.70–12.80)
Long ≥13 to ≤15.5	Deceased	15 (39 %)	14.21* (0.58)	14.15* (13.18–15.19)	14.08* (13.63–14.72)
Exceptional ≥15.6	Deceased	6	15.98 (0.49)	15.80 (15.68–16.96)	
	Alive^a^	5	16.82 (0.65)	17.13 (16.04–17.50)	
	Sub-total	11 (28 %)	16.36* (0.69)	16.04* (15.68–17.50)	16.47* (15.76–NE)
Overall	Overall	39 (100 %)	14.11 (1.86)	14.01 (9.68–17.50)	14.01 (13.18–14.77)

Within each column (mean, median, MST), values which share an asterisk (*) are each significantly different from one another (P < 0.0001) by parametric and non-parametric analysis of variance for age at death/censor date and by Kaplan–Meier survival analysis; values within a column with no asterisk were not compared

*CI* confidence interval, *NE* not estimated using Statistix commercial software

^a^July 31, 2014

Cancer was the reason for euthanasia for 13 dogs (38 %) with 20 dogs (59 %) undergoing euthanasia for other reasons and one dog died overnight (enteritis, colitis and protein-losing enteropathy were found on post-mortem examination) (Table 5). Of the 13 dogs that underwent euthanasia as a result of cancer: 54 % of these dogs lived to an Expected age, 31 % were in the Long group and 15 % in the Exceptional group although these proportions were not significantly different (P = 0.3, Table 5).

**Table 5:** Cause of death for 34 dogs by gender and lifespan category as of 31 July 2014

Cancer	Expected: ≥9 to ≤12.9 years		Long: ≥13 to ≤15.5 years		Exceptional: ≥15.6 years		Total
Female	3 =	Haemangiosarcoma unspecified type/location Haemangiosarcoma splenic with metastasis Lymphosarcoma	3 =	Plasma cell tumour Haemangiosarcoma Osteosarcoma	2 =	Haemangiosarcoma splenic Adenocarcinoma pulmonary	8
Male	4 =	Haemangiosarcoma prostate/bladder Prostatic cancer Mast cell tumour metastasis Urinary tract cancer—TCC	1 =	Urinary tract cancer bladder	0		5
Sub-total	7 (54 %)		4 (27 %)		2 (33 %)		13 (38 %)
Other							
Female	4 =	Intervertebral disc disease Mega-oesophagus causing chronic vomiting Seizures—unresponsive to medication Osteoarthritis (CHD)	7 =	Gastric dilatation volvulus Chronic kidney disease (asymptomatic)^a^ Progressive seizures Septicaemia Osteoarthritis (2) Quality of life	4 =	Osteoarthritis (2) Quality of life (2)	15
Male	2 =	Osteoarthritis	4 =	Found deceased in kennel (enteritis, colitis and PLE)^b^ Gastro-intestinal inflammation^c^ Chronic kidney disease (end stage) Myocardial fibrosis	0		6
Sub-total	6 (46 %)		11 (73 %)		4 (67 %)		21 (62 %)
Total	13 (100 %)		15 (100 %)		6 (100 %)		34 (100 %)

33 dogs underwent euthanasia due to deteriorating quality of life and one dog found dead in the morning^b^

^a^Histopathology revealed inflammatory process in kidney indicating chronic infection (asymptomatic)

^b^Post-mortem examination revealed enteritis and colitis with evidence of protein-losing enteropathy and mild multifocal glomerulosclerosis

^c^Histopathology revealed inflammatory changes in gastro-intestinal tract

### Body weight and composition changes

From the start of the study to age 9, body weight increased for all three lifespan groups (Fig. 2) but the changes over this period were not significantly different (Table 6). In contrast, there was a significant change in body weight (kg/dog/year) during the span of 9–13 years as Exceptional dogs increased body weight while the Long lifespan dogs lost weight (+0.53 vs. −0.91 kg/dog/year respectively, *P* = 0.007). The Expected lifespan dogs also lost weight during this period (−0.15) but the loss was not significantly different from the Exceptional or Long lifespan groups. After age 13, dogs in the Exceptional and Long groups both lost a comparable amount of body weight. The polynomial plot in Fig. 2 reveals that the dogs in the Exceptional group had a lower peak in body weight and this peak occurred at a later age compared to the dogs in the Expected and Long lived groups.

**Table 6:** Body weight change (slope) for three age categories of Labrador retrievers up to death/censor date 31 July 2014

Longevity category	Up to 9 years			9–13 years			After 13 years		
	N	Mean (in kg/dog/year)	SEM	N	Mean (in kg/dog/year)	SEM	N	Mean (in kg/dog/year)	SEM
Expected (≥9 to ≤12.9 years)	13	0.59	0.3	13	−0.15	0.31		N/A	
Long (≥13 to ≤15.5 years)	15	0.52	0.28	15	−0.91*	0.28	15	−1.41	0.68
Exceptional (>15.6 years)	11	0.4	0.36	11	0.53*	0.32	11	−1.31	0.74

Means within the 9–13 years column which share an asterisk (*) are significantly different (P = 0.007); none of the means within the other two columns (up to 9 years, after 13 years) show any significant differences among them (P > 0.05)

*N* number of dogs, *SEM* standard error of the mean

The 39 dogs underwent between 3 and 10 DEXA scans during the study period with a median of seven scans per dog. Total fat mass (g/dog/year) increased in all lifespan groups to age 13 but the increase was significantly slower only for the Long lifespan dogs when compared with Expected dogs which accumulated fat at 3.1 times the rate over this time period (slopes of +320 vs. +1000, *P* = 0.003 Table 7). In contrast, all groups lost a similar amount of total lean mass (g/dog/year) through age 13 ranging from −593 (Expected), −461 (Long) to −269 (Exceptional) (*P* > 0.05). Percent body fat increased significantly more slowly to age 13 in both the Exceptional and Long-lived dogs compared with Expected dogs (1.55 and 1.25 vs. 2.69, respectively, *P* = 0.02 and *P* = 0.002). Congruently, the percentage loss of lean mass through age 13 was significantly slower for dogs of Exceptional and Long lifespans when compared with those having an Expected lifespan (−1.58 and −1.31 vs. −2.69, respectively, *P* = 0.02 and *P* = 0.002). Similarly, the change in the fat to lean ratio to age 13 was significantly greater in the Expected dogs versus both the Exceptional and Long lived dogs (*P* = 0.02 and *P* = 0.002). Total bone mineral density (BMD) was significantly lower for Expected compared to Exceptional and Long (*P* < 0.04). There were no statistically significant differences among the lifespan groups for changes in total bone mineral content (BMC) *(P* = 0.2). There were no statistically significant differences in BCS slopes over time among the three lifespan groups (data not shown).

**Table 7:** Results of linear mixed regression for average changes in body composition determined by DEXA scans performed annually up to 13 years of age

DEXA variable	Longevity category								
	Expected (≥9 to ≤12.9 years)			Long (≥13 to ≤15.5 years)			Exceptional (≥15.6 years)		
	N	Mean^a^	SEM^b^	N	Mean^a^	SEM^b^	N	Mean^a^	SEM^b^
Total fat mass (g)	13	1000*	165	15	320*	152	11	625	170
Total lean mass (g)	13	−593	127	15	−461	80	11	−269	165
Body fat (%)	13	2.69*^#^	0.35	15	1.25*	0.31	11	1.55^#^	0.36
Body lean (%)	13	−2.69*^#^	0.33	15	−1.31*	0.29	11	−1.58^#^	0.34
Fat:Lean^b^	13	0.06*^#^	0.01	15	0.03*	0.01	11	0.04^#^	0.01
Total BMC (g)	13	10	3.2	15	18	3.1	11	16.6	3.2
Total BMD (g)	13	0.0061*^#^	0.0022	15	0.0123*	0.0019	11	0.0131^#^	0.0024

Means within a row that share an asterisk (*) or a hash (#) are significantly different (P < 0.05); means within a row with no asterisk or hash are not significantly different (P > 0.05)

*DEXA* Dual-energy x-ray absorptiometer scans obtained using DXA Model Delphi-A, Serial No. 70852; Bedford, MA, USA

*N* number

^a^ Means and standard errors of the mean (SEM) for changes in body composition (slope) as g/dog/year or %/dog/year up to 13 years

^b^ The fat to lean ratio was determined as total fat mass (g)/total lean mass (g)

## Discussion

The results of this longitudinal study show that a greater than expected proportion of Labrador retrievers lived well beyond that of the expected breed lifespan as nearly 90 % (n = 35) of the dogs met or surpassed the consensus average life expectancy for the breed of 12 years and 28 % (n = 11) went on to achieve Exceptional longevity (≥15.6 years). In spite of being 16 and 17 years of age, the five dogs remaining alive at the end of the study continued to be full of life, active, social and highly engaged with their animal welfare specialists and social groups according to the independent veterinarians and Labrador retriever enthusiasts who interacted with these dogs in June 2014. This supports our findings that an increase in healthspan was present in this study population. These findings are similar to those reported in an earlier longitudinal study of the effect of calorie restriction in Labrador retrievers where the ‘Restricted’ group experienced a median lifespan of 13.0 years with a maximum lifespan of 14.5 years compared to 11.2 and 13.2 years, respectively, for the ‘Control’ group [2, 19, 25, 26]. It may therefore be suggested that identifying the appropriate calorie balance (i.e. calorie intake that matches calorie expenditure) in order to reach and maintain an ideal BCS throughout the lifespan of Labrador retrievers and adhering to this feeding level may be essential in achieving a long, healthy life.

There was a slower loss of total and significantly slower loss of percentage lean body mass in the Exceptional group versus the Expected group together with a slower increase in total body fat and a significantly slower increase in percentage body fat and a significantly lower change in fat to lean ratio. This is similar to the findings of another study in which body composition was measured from 6 years of age where the mean total lean mass was significantly greater in the ‘Control’ group compared to the ‘Restricted’ group; additionally, there was a progressive decline in total lean mass after 9 years of age in the ‘Control’ group but not in the ‘Restricted’ group until after 11 years of age [2, 19]. Furthermore, although mean percentage lean mass decreased significantly in both groups from 6 to 12 years of age it was always significantly higher among the ‘Restricted’ dogs than the ‘Control’ dogs [19]. Mean total and percentage body fat mass increased significantly in both groups from 6 to 12 years of age and was always significantly higher in the ‘Control’ group. There was no correlation between total lean and fat mass in the ‘Restricted’ group and this may suggest underlying processes that drive the beneficial longevity response in these dogs may be multiple and driven independently [2]. Somewhat paradoxically, the dogs in the Long lifespan group of the current study lost weight between the ages of 9 and 13 years whilst the Exceptional lifespan dogs maintained or slightly gained weight during this time period. Body mass and body composition are related to an individual dog’s size and they may also independently influence the rate of ageing and longevity. The risks of an increase in the incidence and severity of chronic disease associated with high body fat has been reported in other studies [27–29]. In adult nonhuman primates, lower morbidity rates also have been reported in studies comparing calorie-restriction versus ad lib feeding. In the calorie-restriction primates (fed 30–40 % less calories than their ad lib pair mate) the body fat content was within the normal range of 10–22 % [30]. Typically, the body fat in ad lib fed primates can range from 17–44 % [31]. This calorie-restriction induced prevention of morbidity does not therefore require excessive leanness.

A comparison between the dogs in the current study and those from the calorie restricted study [2, 19, 25] shows that energy intake per unit of body weight kcal/kg/day for the ‘Restricted’ group (≈46.5) was very close to the 46.2–48 kcal/kg/day of dogs in the current study (Table 8). Therefore, on an energy intake basis, we can consider these groups of dogs to be similar. This is reflected by the ‘Restricted’ group having a BCS in the ideal range of 4–6 on a 9-point scale from 6 to 12 years of age. However, the oldest dog in the ‘Restricted’ group died at 14.5 years of age whilst a female in the current study was still alive on July 31, 2014 at 17.5 years of age. We can only speculate why we had dogs showing much longer lifespan than the dogs in the other study despite being on the same calorie intake and BCS level. The detailed data on body composition is providing a potential in investigating reasons related to increased healthspan beyond the BCS and calorie intake. As well, the overall median lifespan was 13.0 years for the 48 dogs in the calorie restricted study whilst the median age (at death and for those still alive) was 14.01 years for the 39 dogs reported here. One important difference between the Labradors reported in this study and those in the calorie restricted study is that all the dogs in the present study were kept at a BCS between two and four throughout the study. The classic calorie restriction model used to examine the effects of feeding on the ageing process is often described as one of undernutrition without malnutrition. This usually involves control animals that are fed ad libitum (free choice with no or little restriction) as in the studies by Kealy, Lawler et al. [2, 19, 25, 26] and comparing these to animals fed a set restricted number of calories (i.e. not free choice). However, the energy intake of such ‘control fed’ animals often significantly exceeds the amount expended, resulting in a substantial gain in body weight, or positive energy balance, which is often associated with the early onset of disease [19, 32–34]. As the majority of dogs were recruited from the same breeder, the variation in environmental exposures among these dogs is reduced even if those dogs did not share the exact same habitat (for example due to temporal differences among litters). With regards to the genetic background, the dogs in this study were not as closely related as the dogs in the restricted feeding study. We recognise that the longevity effect seen in this study could be partly or wholly explained by the relatedness/line breeding of the dogs. However, dogs from the one breeder were found in all three longevity categories so although there might be a genetic predisposition for longevity, not all dogs expressed this characteristic.

**Table 8:** Energy intake of Labrador retrievers in the current study and a previous calorie restricted study

Average daily intake of energy^b^	Current study			Calorie ‘restricted’ group^a^
	Expected	Long	Exceptional	
MJ/day	5.88	5.84	5.56	5.15
kcal/day	1405	1397	1329	1230
Energy intake kcal/kg/day	46.2	46.2	48	≈46.5

^a^Group of Labrador retrievers fed 25 % less than their ‘Control’ fed pair; Kealy et al. [19, 25] and Lawler et al. [2]

^b^Metabolic energy of the test diet fed was 3669 kcal/kg based on a Modified Atwater calculation

As the mean body weights for the three longevity groups at the start of the study were not significantly different, we suggest that body weight alone, and particularly during middle age (5–8 years) is not predictive of longevity. The association over time between changes in different body mass components and health may provide key insights into future recommendations on how to manage our ageing dogs more successfully to achieve an improved healthspan. One key body mass component is skeletal muscle. A number of age-related changes may contribute to the gradual age-related loss of skeletal muscle which is reported in humans and dogs [35]. This loss of lean body mass in the absence of disease is termed ‘sarcopenia’ and it is important to differentiate this from ‘cachexia’ which is the loss of lean body mass with disease [36]. Sarcopenia is defined in humans as “a syndrome characterised by progressive and generalised loss of skeletal muscle mass and strength with a risk of adverse outcomes such as physical disability, poor quality of life and death” [37]. Sarcopenia in humans usually begins early in life and between the ages of 20 and 60 years there is on average a loss of 40 % of lean muscle mass [38]. In sarcopenia, the loss of lean body mass often is accompanied by an increase in fat mass so the total weight may not change (or may even increase), thus masking the sarcopenia [36]. Previous studies in dogs have shown that a high percentage of lean mass was associated with a protective effect from death whilst declining grams of lean body mass as well as a high percentage and absolute (grams) of fat mass predicted death [26]. The present study is the first published study to show an association between a slower rate of loss of lean body mass and exceptional longevity in dogs. The polynomial smooth plot (Fig. 2) revealed that the oldest dogs in the Exceptional group had an initial peak in body weight at 12 years of age and then re-gained weight after 16 years of age following a period of weight loss. This weight re-gain could represent a longevity advantage for these ‘oldest of the old’ dogs, however due to the small number of dogs included in this category, results need to be interpreted with caution. Further evaluation of what contributes to this late weight gain (e.g. slower loss of lean body mass combined with an increase in fat mass) could provide information on preferable body composition changes that would confer a longevity advantage to dogs.

Among human centenarians, women outlive men by four to one and, like women, female Rottweilers were more likely to achieve exceptional longevity (age at death ≥13 years) than males of this breed in a large retrospective study that examined the effect of ovary exposure up to 8 years of age [16]. However, this survival advantage was lost if the dogs underwent ovariectomy during the first 4 years of life. In females that retained their ovaries for more than 4 years, the likelihood of reaching exceptional longevity increased to more than three times that of males. Additional data from female Rottweiler dogs revealed that the number of years of ovary exposure was associated with exceptional longevity [39]. Females with the longest ovary exposure (6.1–8.0 years) were 3.2 times more likely to reach exceptional longevity than females spayed during the first 2 years of life (P = 0.002). Furthermore, there was no evidence of a female’s physiological investment in offspring (number of litters, total number of pups, age at first reproduction, mean inter-birth interval, age at last reproduction) being associated with a reduced longevity [18]. These findings suggest that the ovaries participate in functions beyond reproduction which may include a role in the orchestration of exceptional longevity. Contrastingly, there was no effect of gender or the age at neutering on the risk of death for the dogs in the present study. The discrepancies between the Rottweiler study and our study may be related to the small sample producing a low statistical power as supported by the finding that whilst female Labrador retrievers tended to live longer than males, there was no statistically significant effect of gender on risk of death and age at death.

Whilst these dogs were not housed in a natural pet environment (such as in a household with human ‘companions’), they were housed in compatible social groups and also had regular daily interaction with people much as a household pet might. Although there may be large differences between the structured management of the study dogs and typical household/family dogs, the benefit of keeping the study dogs under very similar conditions throughout the study removes the effect of many potential confounding variables that could change over time in a household environment. A study of family-owned dogs would require a much larger sample size when investigating associations between risk factors and outcomes. Such a large sample would not only be very expensive but is likely to suffer from participants leaving the study due to the long time commitment. The decision not to treat any cancer diagnosis with chemotherapy or radiotherapy, and to manage the dog through palliative care with other medications, was due to an ethical position by the company as well as representing a choice that dog owners might make if their own pet was diagnosed with cancer, particularly at an older age. All dogs with cancer and other chronic medical conditions had quality of life assessments initiated at the time of diagnosis to ensure that their health and well-being was managed ethically and appropriately (see footnote 4). Other medical issues such as ear and skin conditions were managed by the healthcare staff under veterinary supervision. The radiation risk associated with the annual general anaesthetic for the DEXA scans was assumed to be negligible based on published information about DEXA scans in people [40].

Conducting breed-specific longevity studies is both a strength and a limitation; initially, it may seem to potentially limit the understanding of healthy ageing. For instance, it has been shown that the median lifespan of crossbreds is greater than purebred dogs [12, 41] and that even within a set weight range of breeds there can be differences in the median age of death [8, 12]. However, by evaluating a single breed, rather than multiple breeds, then the influence on longevity by factors such as obesity and ovary exposure, as well as the age at onset of specific age-related disease might become clearer [42] as variation on other factors is reduced. When multiple breeds are evaluated together, such associations may be disguised or distorted. A limitation of this study is the absence of a control group fed a different diet as a result of a business decision in 2008 to discontinue the third study group. However, the strength of the cohort approach taken in this longitudinal study is that it allows further work using survival analysis to examine the effects of the time-varying measurements on longevity and development of disease. Whilst this was not a birth cohort and the results may be confounded by early life experiences, previous work has shown that deaths in Labrador retrievers less than 6 years of age were mostly due to trauma [43] and in the aforementioned calorie restriction study only two dogs from the original 48 died before 6 years of age [26]. There are many factors that may have contributed to the ability of these dogs to exceed a typical lifespan and reach exceptional longevity. These include genetics, husbandry, preventative healthcare, socialisation, housing and environmental enrichment. Longevity is generally accepted as having a heritability of approximately 25–30 %, with the effect of the environment having a much larger influence [44]. Nutrition is part of this environmental influence and therefore has a potentially significant role to play in longevity. Many of the probable environmental influences that could impact successful ageing and longevity were maintained as constant as possible for all the dogs in the current study. The nutritional matrix fed to all dogs incorporated key nutritional components that have been shown to benefit canine health and well-being based on results of short-term research studies. This longitudinal study is the first to incorporate all of these nutritional components into the same dietary matrix consistently fed over an extended period of time. As dogs pass through the ageing process their nutritional requirements change reflecting a normal response to age-related metabolic and physiologic responses. The dietary matrix fed was designed to address recognised changes that take place as dogs pass from adulthood into their senior years. The test diet (Table 2) was a matrix based on animal protein ingredients, balanced omega-6/3 fatty acids with a blend of low glycaemic index carbohydrates alongside L-carnitine. Antioxidants vitamin E and beta-carotene, the moderately fermentable beet pulp and the prebiotic fructooligosaccharide were also incorporated. Finally, the outside of the dry food was coated with sodium hexametaphosphate to help with reduction of tartar build-up. It is likely that there are many dietary factors including total energy intake relative to energy needs, specific nutrients and other non-nutrient bioactive substances that, individually or collectively, influence the ageing process.

Highly successful ageing can be considered as being robust in an age-specific way and which translates into being resilient to disease, including cancer. For humans it has been hypothesised that centenarians either markedly delay or escape age-associated morbidity such as heart disease, stroke, diabetes, cancer, and Alzheimer’s disease [45]. Consistent with this idea is the proposition from James Fries, known as the compression of morbidity hypothesis which states that individuals who reach the limits of human lifespan compress the onset and duration of illnesses toward the end of life [46, 47]. This hypothesis predicts that, in order to achieve their extreme old age, centenarians markedly delay or even escape diseases that would otherwise be lethal at younger ages. A retrospective cohort study of 424 human centenarians examining the ages of onset for 10 age-associated diseases and excluding cognitive impairment, found that the centenarians fitted into three morbidity profiles; Survivors, Delayers, and Escapers [48]. Survivors (38 % of study population) had a diagnosis of an age-associated illness prior to the age of 80, Delayers (43 %) were individuals who delayed the onset of age-associated illness until at least the age of 80 and Escapers (19 %) were individuals who attained their 100th year of life without the diagnosis of common age-associated illnesses. Centenarians have, therefore, shown successful ageing and have in some way developed an age-related robustness and resilience to disease. This resilience includes cancer, where proportional mortality rates increase with age during most of adulthood but decline in advanced age from nearly 40 % of all deaths between the ages of 50 and 69 to only 4 % of all deaths in patients older than age 100 years [49]. These data suggest that the oldest-old humans have a cancer-resistant phenotype compared to the general population. In the current study, cancer was the cause of euthanasia in 54 % of the dogs that lived to an Expected age, 27 % in the Long group and 33 % in the Exceptional group, suggesting that the longer-living dogs may have been less likely to experience cancer although this was not statistically significant. We were not able to show any association of cause of death with lifespan group or gender although this is likely due to the small numbers of dogs in each group.

## Conclusions

The findings of this study indicate that the life-long maintenance of lean body mass and an attenuated accumulation of body fat are key factors influencing successful ageing as reflected by longer healthspan. In the current study, the combination of a high quality plane of nutrition with appropriate husbandry and veterinary care resulted in 28 % of the dogs reaching an Exceptional lifespan of ≥15.6 years and almost 90 % of the dogs exceeded the typical lifespan of 12 years. Future work includes further analysis of the data from this 10+ year study using survival analysis and other techniques to look at how variables change over time. The long-term objective is to provide clear and practical recommendations for both dog owners and veterinarians so that all dogs can live to their full genetic potential.

## Abbreviations

BCS: body condition score
BW: body weight
C: celsius
Cox: cox proportional hazards regression
DEXA: dual-energy x-ray absorptiometer
IACUC: Institutional Animal Care and Use Committee
KM: Kaplan–Meier survival analysis
MH: mannoheptulose
MST: median survival time
P&G: Procter & Gamble Pet Care

## Footnotes

1 The IACUC board had both internal and external board members. The external members were represented by a local High School teacher, and a local veterinarian from Dayton (OH, USA) with research experience but with no affiliation with any type of research institute.

2 International Animal Welfare Advisory Board annual report 2013 is available at https://www.iams.com/iams-truth/international-advisory-board-activities.

3 Dogs were housed in indoor kennels either on their own or in pairs. The kennels were 122 × 122 cm (single) or 244 × 244 cm (double) and arranged either side of an open corridor allowing dogs to see and hear other dogs in the group. Suitable bedding (Kuranda Dog Beds, Glen Burnie, Maryland, USA) was provided for each dog. The kennels were made from welded stainless steel mesh and polycarbonate panels. The floors were epoxy and were heated. A two-way door led from each kennel to a partially covered outdoor run (365 × 243 cm) which provided additional access to natural light. All runs had concrete flooring and dogs had free access to their run throughout the day and night.

4 Assessments were conducted by animal welfare specialists, animal husbandry staff and healthcare staff (attending veterinarian, veterinary nurses) to assess quality of life using a 10-point Likert scale ratings to assess food/water intake (hunger/thirst), pain/discomfort, mobility, hygiene, happiness and number of “good days”. When the dog was having more “bad days” than “good days” then the attending veterinarian convened a panel to discuss an end-of-life decision; original concept, Oncology Outlook, by Dr. Alice Villalobos, Quality of Life Scale Helps Make Final Call, VPN, 09/2004; scale format created for author’s book, Canine and Feline Geriatric Oncology: Honouring the Human-Animal Bond, Blackwell Publishing, 2007. Revised for the International Veterinary Association of Pain Management (IVAPM) 2011 Palliative Care and Hospice Guidelines.

5 Eukanuba^®^ Senior Maintenance Dog Food. For the study period reported this product was owned and manufactured by Procter & Gamble, Cincinnati, Ohio, USA.

6 Statistical software: SAS version 9.3 (SAS Institute, 2011, Cary, NC, USA), Statistix version 10 (Analytical Software, 2013, Tallahassee, FL), SPSS version 22 (IBM SPSS, 2013, Chicago, IL, USA) and Stata version 13.1 (StataCorp, 2014, College Station, TX, USA).
